# Sex-Associated Cerebellar and Hippocampal Volume Reduction in Alzheimer’s Disease: Insights from the Clinical ADNI Cohort and STZ Animal Model

**DOI:** 10.3390/ijms26104810

**Published:** 2025-05-17

**Authors:** Krista Mineia Wartchow, Leticia Rodrigues, William Jones Dartora, Regina Biasibetti, Nicholas Guerini Selistre, Artur Lazarian, Carmen Barrios-Castellanos, Nicholas Bartelo, Carlos-Alberto Gonçalves, Laura Beth J. McIntire, on behalf of Alzheimer’s Disease Neuroimaging Initiative (ADNI)

**Affiliations:** 1 Brain Health Imaging Institute, Department of Radiology, Weill Cornell Medicine, New York, NY 10065, USA; wjd4002@med.cornell.edu (W.J.D.); arl7014@med.cornell.edu (A.L.); crb4005@med.cornell.edu (C.B.-C.); nib4003@med.cornell.edu (N.B.); lbm7002@med.cornell.edu (L.B.J.M.); 2 Biochemistry Post-Graduate Program, Federal University of Rio Grande do Sul (UFRGS), Porto Alegre 90035-003, Brazil; letigues@gmail.com (L.R.); regina.biasibetti@gmail.com (R.B.); nicholas.gueriniselistre@gmail.com (N.G.S.); casg@ufrgs.br (C.-A.G.)

**Keywords:** Alzheimer, ADNI, GFAP, cerebellum volume, hippocampus volume, sex

## Abstract

While the greatest risk factor for Alzheimer’s disease (AD) is aging, women are disproportionately affected by the disease. Interestingly, the hippocampus and cerebellum exhibit gender-specific cytoarchitecture differences, which are associated with AD, despite the absence of a role in animal reproductive behavior or hormonal signaling. This study investigates the potential association of sex differences associated with AD by interrogating cerebellar and hippocampal volume in preclinical (MCI) as well as clinical phases of AD compared to cognitively normal patients (CN) and in an animal model of AD, the streptozotocin (STZ)-induced sporadic AD model. In order to investigate putative changes in cerebellum and hippocampus in a rat model of AD, we used a STZ-induced sporadic AD model at three different time points (2, 4, and 8 weeks) after surgery in male and female rats. Previous studies have reported hippocampal-dependent changes as well as sex-dependent behavioral and signaling effects in the STZ animal model of sporadic AD while our current study showed involvement of cerebellum-mediated changes. To interrogate the role of cerebellar volume in AD progression within the human context, we analyzed data available through the Alzheimer’s Disease Neuroimaging Initiative (ADNI). In a cross-sectional analysis, we observed that levels of peripheral Glial Acidic Fibrillary Protein (GFAP) (astrocytic protein) were associated negatively with cerebellar and hippocampal volumes (β = −0.002, *p*-value = 0.04; β = −6.721, *p*-value < 0.0001) and were associated with sex specific differences in males. Our analysis identified that the effect on hippocampal volume was earlier in disease stage, reinforcing the relevance of longitudinal alterations of cerebellum and hippocampus volume over time. The STZ animal model of sporadic AD, corroborated the progressive changes in hippocampal volume and more minor and temporally delayed involvement of the cerebellum volume changes which were dependent on sex. This suggests that cerebellar involvement may be secondary to hippocampal neurodegeneration, and both regional differences were dependent on sex. Due to the association with GFAP, our findings may be due to network astrocyte connection spread regardless of primary pathology. Overall, our study uncovers a novel role for cerebellum in AD in a model and in the human context.

## 1. Introduction

Alzheimer’s disease (AD) is one of the most debilitating neurodegenerative disorders, affecting middle-aged and elderly individuals with higher prevalence in those over 65 years of age [1]. AD is characterized by familial and sporadic etiologies of which sporadic AD comprises approximately 95% of cases and results from a complex progression over decades [2]. Histopathologically, there is evidence of extensive neuronal loss as well as senile plaques formed by the extracellular deposition of amyloid β peptides and intracellular neurofibrillary tangles [3]. In addition, a series of events that lead to neuronal dysfunction, especially in cholinergic neurons, occur in areas related to cognition and memory formation, such as the prefrontal cortex and hippocampus, along with associated connectivity networks. Thus, neurochemical imbalance and functional network defects are concurrent with neurodegeneration [4].

Importantly, sex differences are apparent in AD, with women comprising two-thirds of the population affected by the disease. Disease progression is also sex dependent with males showing a more accelerated progression in contrast to less severe progression in females, who live longer with the disease [5]. Several brain areas involved in the regulation of sexual behavior differ in cytoarchitecture between males and females, such as the hypothalamic ventromedial nucleus and the stria terminalis bed nucleus, which are more extensive in males [6]. Interestingly, the hippocampus and cerebellum are not primarily involved in the reproductive behavior or hormonal regulation, but also show sexual dimorphism [7]. The hippocampus plays a crucial role in memory and spatial orientation, concurring with studies that suggest spatial orientation in humans may be associated with sexual dimorphism. The cerebellum is also responsible for cognitive tasks, including language, working memory, and spatial processing [8]. Importantly, patients with AD present alterations in structure and metabolism of the cerebellum [9,10]. This region is also involved in diseases such as autism and attention deficit disorder, which have differential prevalence based on sex, suggesting that there is also sexual dimorphism in these various disease mechanisms [11].

Multiple animal models have been designed to investigate mechanisms of AD etiology, progression, and potential therapeutics. Most of them are transgenic mouse models created by overexpressing human APP, PS1, and/or tau genes [12]. However, these models do not replicate the development of sporadic AD (sAD) due to the constitutive over-expression of these proteins. The primary incidence of AD in humans is sporadic and develops after age 65 [1]. Furthermore, the leading cause of AD involves the interaction between several genetic and others etiologic factors [13]. While age is considered the main risk factor [14,15], there are other potential risks such as hypertension, cholesterol/apolipoprotein alleles, head trauma, inflammation, environmental, and metabolic (diabetes), more specifically glucose and insulin [16]. A commonly used non-transgenic model for sAD is the intracerebroventricular (ICV) infusion of streptozotocin (STZ), which produces a similar characteristic pathology such as altered glucose metabolism, insulin signaling, and synaptic dysfunction [17,18,19]. Insulin resistance in the central nervous system (CNS) has been identified as one of the early events contributing to the onset of sAD; it is involved in neuropathology [20,21], and insulin administration to the CNS has been shown to be effective in ameliorating cognitive impairment in early AD [22].

Previous studies of glucose utilization using Fluorodeoxyglucose Positron Emission Tomography (FDG-PET) found no difference in cerebellar glucose uptake between AD and Cognitively Normal (CN) participants, which led to its adoption as a normalizing area for standardized uptake values (SUVs) [23,24]. However, with the advent of extensive standardized neuroimaging studies such as the Alzheimer’s Disease Neuroimaging Initiative (ADNI), full volumetric changes in the cerebellum are currently under investigation. Although β-amyloid accumulation and tau aggregates are thought to be involved in neurodegeneration ultimately leading to differences in topological patterns and progression of AD-related volumetric changes, regional brain atrophy in AD does not follow either β-amyloid or tau topological patterns precisely [25,26]. Thus, progression-dependent volumetric changes in the cerebellum may represent neuronal loss and potentially astrocyte hypofunction in the cerebellum in the absence of amyloid and tau pathologies [27].

Astrocytes are the most abundant cells in the brain tissue and are involved in several functions, including maintenance of the brain blood barrier and metabolic support to neurons [28]. The glial fibrillary acidic protein (GFAP) is a primary biomarker for their identification and for AD progression [29]. The astrocytes take up glucose and are involved in multiple metabolic energy pathways, such as neurotransmitter and glutathione (GSH) synthesis and lipid metabolism [30]. In the CNS, GSH is a key antioxidant defense against oxidative damage derived from astrocyte glutamate metabolism [31].

The involvement of the hippocampus in AD is already well-described and has been modeled using the STZ model, which results in hippocampal volume changes both in sexes and overtime [32]. In addition, cerebellar involvement in the initial cognitive decline is described in several neurodegenerative diseases [33]. Although the cerebellum is reported to be relatively spared from AD associated pathology and changes in glucose metabolism, the volumetric changes in AD are poorly studied. This study aims to provide valuable insights into the cerebellum’s association with neurodegeneration-related changes over time and its potential implications for AD progression in the context of sexual dimorphism. In the human context, our study analyzed changes in cerebellar volume between the sexes during the preclinical (MCI) and clinical phases of AD compared to cognitively normal (CN) participants. These changes were also demonstrated in the STZ–ICV animal model, emphasizing our findings’ translational relevance.

## 2. Results

### 2.1. Animal AD Model Phase

#### 2.1.1. Sexually Dimorphic Cerebellar Response to STZ

**Figure 1:**
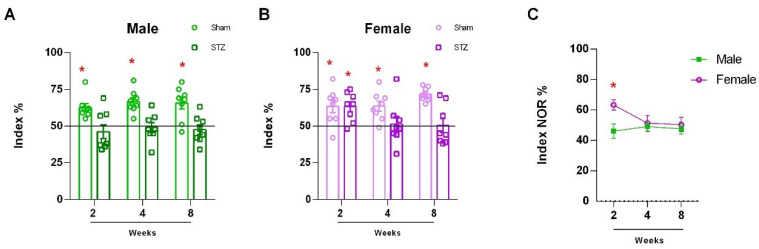
NOR performance of rats submitted to ICV-STZs-AD model. Male (**A**) and female (**B**) rats were submitted to bilateral ICV injection of STZ. Two, four, or eight weeks later novel object recognition (NOR) was performed. (**A**) NOR index of the male rats 24 h after training trial. (**B**) NOR index of the female rats 24 h after the training trial. For the NOR test, each point represents the mean ± standard error. * Significant differences were detected by comparing STZ and sham group (N = 8–9, One sample *t*-test difference to 50%, *p* < 0.05). (**C**) Gender-related differences in the cerebellum of rats submitted to ICV-STZ infusion. Comparison over time between males and females in the STZ model. Values are mean ± standard error. * Significant difference between male and female groups of the related time (N = 7–9, two-way ANOVA followed by Sidak’s test, *p* < 0.05).

We confirmed previous results from [32], for the effect of STZ on learning and memory, in the novel object recognition (NOR) paradigm, which was used to evaluate the learning and memory performance of male and female rats 24 h after training (long-term memory). As previously reported [32], after two, four, and eight weeks of STZ (or vehicle) injection, males presented a significant impairment in long-term memory recognition (Figure 1A: Sham 2 W, *p* = 0.0021; 4 W, *p* = 0.0005, and 8 W, *p* = 0.0066; STZ 2 W, *p* = 0.4336; 4 W, *p* = 0.7729 and 8 W, *p* = 4678). However, females presented resistance to STZ deficits in NOR and a delayed response, presenting memory impairment only after four and eight weeks of STZ-injection (Figure 1B: Sham 2 W, *p* = 0.0179; 4 W, *p* = 0.0056, and 8 W, *p* < 0.0001; STZ 2 W, *p* = 0.0065; 4 W, *p* = 0.8163 and 8 W, *p* = 9396). In order to determine if sex dependent differences were significantly dependent on time after SZT ICV infusion, we performed two-way ANOVA followed by Sidak’s test, and we found significantly different preference index % in females which were delayed or not affected by SZT ICV infusion compared to males. Interestingly, males showed continuous behavioral impairment in NOR starting in the second week after STZ treatment, in contrast to females, which showed a reduction only after the fourth week (Figure 1C, 2 W, *p* < 0.0001; 4 W, *p* = 0.9977 and 8 W, *p* = 0.9158).

Previously, we reported astrocytic changes in the hippocampus which were concurrent with STZ induced deficits in learning and behavior in the NOR test at 2 h and 24 h resulting in the same late memory impairment in males and females [32]. To investigate the possible mechanisms underlying the sexually dimorphic behavior in NOR after to STZ treatment in this study, we investigated neuronal neurotransmitter function and metabolism in cerebellar tissue which has been shown to harbor sexual dimorphism [7].

**Figure 2:**
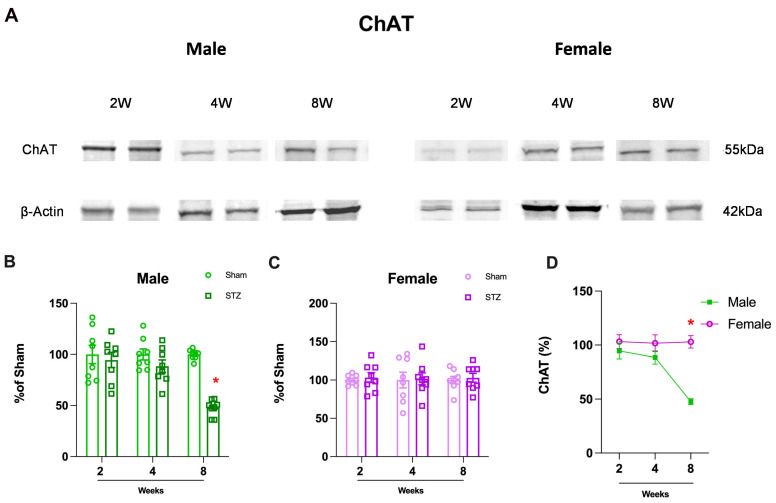
Choline acetyltransferase (ChAT) content in Cerebellum of rats submitted to ICV-STZs-AD model. (**A**) Representative Western blot images. (**B**) Male and (**C**) female rats received bilateral ICV injections of STZ. After 2, 4, or 8 weeks (2 W, 4 W, or 8 W), the cerebellum was dissected, and ChAT levels were analyzed by Western blot. Protein bands were quantified by densitometry. Values are mean ± standard error. * Significant difference between STZ and respective sham group (N = 8, Student’s *t* test, *p* < 0.05). (**D**) Gender related differences in the cerebellum of rats submitted to ICV-STZ infusion. Comparison over time between males and females in the STZ model. Values are mean ± standard error. * Significant difference between male and female groups of the related time (N = 7–9, two-way ANOVA followed by Sidak’s test, *p* < 0.05).

In order to study neuronal function, we first focused on the cholinergic neurons, which have previously been implicated as vulnerable in AD neurodegeneration. Choline acetyltransferase (ChAT) is a key enzyme for the synthesis of acetylcholine in neurons and loss of ChAT may represent loss of cholinergic function or loss of neurons which has previously been associated with AD. We found a significant decrease in ChAT in males (Figure 2B: 2 W, *p* = 0.6443; 4 W, *p* = 0.1802 and 8 W, *p* < 0.0001) but not in females, (Figure 2C: 2 W, *p* = 0.6472; 4 W, *p* = 0.8962 and 8 W, *p* = 0.7091) 8 weeks after the STZ infusion. This loss in ChAT occurs after initial changes in learning and memory, which were observed after 2 weeks. Interestingly, we found that sex-dependent differences were time dependent, and females did not show a loss in ChAT (Figure 2D: 2 W, *p* = 0.7047; 4 W, *p* = 0.3737 and 8 W, *p* < 0.0001).

**Figure 3:**
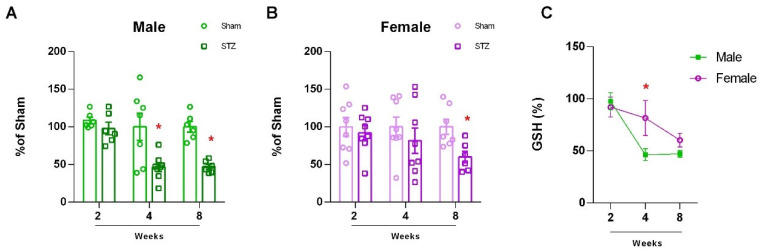
Glutathione levels in Cerebellum of rats submitted to ICV-STZ infusion. Male and female rats were submitted to ICV infusion of STZ. Two, four, or eight weeks later, the male (**A**) and female (**B**) cerebellums were dissected, and levels of GSH were measured by fluorescence emission. Values are mean ± standard error. * Significant difference between STZ and respective sham group (N = 8–9, Student *t* test, *p* < 0.05). (**C**) Gender-related differences in the cerebellum of rats submitted to ICV-STZ infusion. Comparison over time between males and females in the STZ model. Values are mean ± standard error. * Significant difference between male and female groups of the related time (N = 7–9, two-way ANOVA followed by Sidak’s test, *p* < 0.05).

In order to assess oxidative stress in cerebellar tissue, which reflects both neuronal and glial oxidative states, we quantified the tripeptide glutathione, the main antioxidant in the CNS. In males, there was a significant decrease in the group treated with STZ, 4 and 8 weeks after exposure (Figure 3A: 2 W, *p* = 2721; 4 W, *p* = 0.0106, and 8 W, *p* < 0.0001). In females, we observed differences only eight weeks after the STZ model (Figure 3B: 2 W, *p* = 0.6189; 4 W, *p* = 0.414 and 8 W, *p* = 0.0110). Similarly to our findings with ChAT, we observed a delayed loss of GSH content in males and females, which was significantly less at 4 weeks (Figure 3C: 2 W, *p* = 0.9742; 4 W, *p* = 0.0387 and 8 W, *p* = 0.7629).

**Figure 4:**
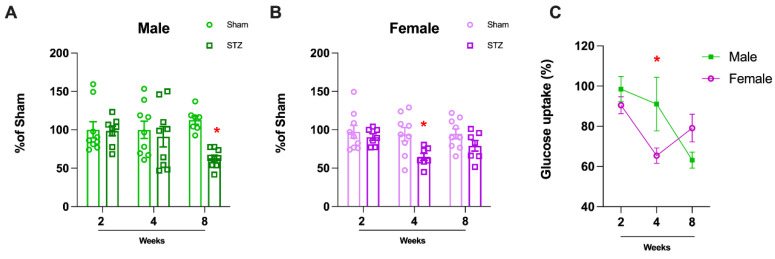
Glucose uptake in Cerebellum of rats submitted to ICV-STZ infusion. Male (**A**) and female (**B**) rat’s cerebellum were dissected, and the glucose uptake assay was performed in acute cerebellar slices. Values are mean ± standard error. * Significant difference between STZ and respective sham group (N = 8–9, Student t test, *p* < 0.05). (**C**) Gender-related differences in the cerebellum of rats submitted to ICV-STZ infusion. Comparison over time between males and females in the STZ model. Values are mean ± standard error. * Significant difference between male and female groups of the related time (N = 7–9, two-way ANOVA followed by Sidak’s test, *p* < 0.05).

To assess glucose uptake in the cerebellum that primarily reflects glial metabolism [34,35], we performed a glucose uptake assay in acute cerebellar slices. There was only a significant difference in males at the later time point 8 weeks after STZ infusion (Figure 4A: 2 W, *p* = 0.9096; 4 W, *p* = 0.6155, and 8 W, *p* < 0.0001). In females, a significant difference was observed four weeks after the STZ infusion, but glucose uptake returned to normal levels after eight weeks (Figure 4B: 2 W, *p* = 0.5097; 4 W, *p* = 0.0165 and 8 W, *p* = 0.1231). Therefore, we observed sex-dependent differences over time, in which males and females displayed different patterns. Namely, females recovered and returned to normal glucose uptake after 8 weeks of the STZ infusion while glucose uptake in males remained reduced over time (Figure 4; 2 W, *p* = 0.8633; 4 W, *p* = 0.0456 and 8 W, *p* = 0.3847).

#### 2.1.2. GFAP Is Altered in Female Rats After STZ Infusion

**Figure 5:**
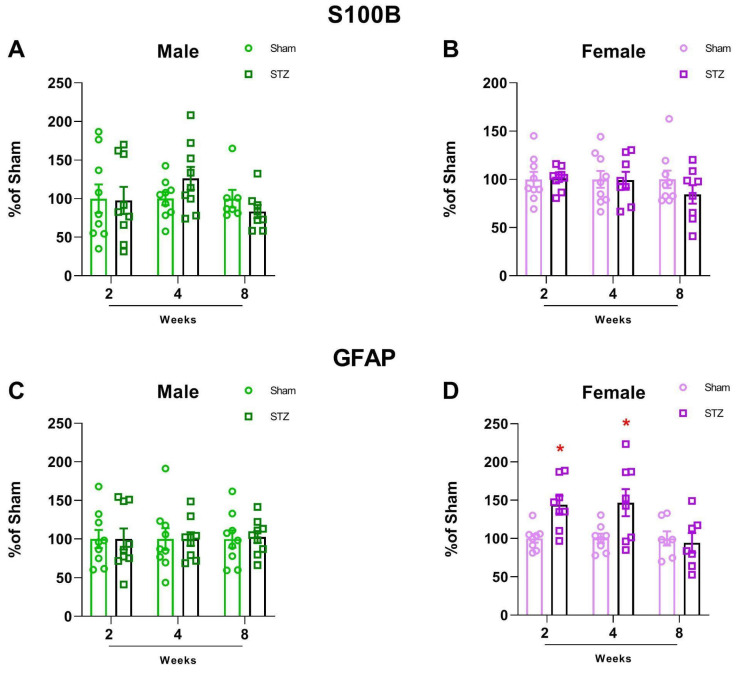
S100B and GFAP content in the cerebellum of rats submitted to ICV-STZ infusion. Male and female rats were submitted to ICV infusion of STZ. Two, four, or eight weeks later, cerebellums were dissected out and levels of S100B (**A**,**B**) and GFAP (**C**,**D**) were measured by ELISA. Values are mean ± standard error. * Significant difference between STZ and sham group of the related time (N = 7–9, *t*-test, *p* < 0.05).

Because the changes in glucose metabolism and oxidative stress may be due to neuronal or glia changes, we then evaluated the astrocyte levels of GFAP and S100B in the cerebellum in order to evaluate astrocytic content levels. In contrast to a previous report that found increased GFAP after STZ treatment at all time points in the hippocampus in both males and females [32], we found a sexually dimorphic increase in GFAP only in females at 2 and 4 weeks after STZ treatment (Figure 5C: 2 W, *p* = 0.999; 4 W, *p* = 0.9916 and 8 W, *p* = 0.8396. Figure 5D: 2 W, *p* = 0.0039; 4 W, *p* = 0.0260 and 8 W, *p* = 0.7177), which was reversed eight weeks after treatment. Interestingly, the levels of S100B did not change in the cerebellum of either sex at any time point (Figure 5A: 2 W, *p* = 0.9281; 4 W, *p* = 0.1427 and 8 W, *p* = 0.2545. Figure 5B: 2 W, *p* = 0.9001; 4 W, *p* = 0.9573 and 8 W, *p* = 0.02518).

#### 2.1.3. Cross-Sectional Analysis Comparing Cerebellum and Hippocampus Volume with GFAP Plasma Levels in the ADNI Cohort

Because we found a sexually dimorphic difference in behavior and GFAP in the STZ animal model, we wanted to determine the translational significance of these findings in the human brain. The Alzheimer’s Disease Neuroimaging Initiative (ADNI) database reports imaging and fluid biomarkers from CSF and plasma. We analyzed GFAP levels in plasma from 124 participants, which also have hippocampal volume, cerebellar volume measured by MRI. Participants without GFAP, hippocampal volume, cerebellar volume, and our covariates, sex and ApoE4 status, were excluded from the ADNI database based on GFAP data subset. For our analysis, we accounted for the time of the participants’ visit for GFAP plasma collection and the time of the data collection for hippocampus and cerebellum volumes. We conducted a cross-sectional analysis for association between GFAP levels and hippocampal and cerebellar volume in a subset comprising a total of 111 individuals. Because apolipoprotein E (ApoE4) is associated with increased risk for AD and has been indicated in sexual dimorphism [36,37,38], we controlled for carriership of one or two ApoE4 alleles in our analysis. In the data subset analyzed, 64 were non-carriers of the ApoE4 allele and 47 were carriers of the ApoE4 allele (ApoE3/4 or ApoE4/4). There was a similar distribution of ApoE4 in males and females: 48.4% of non-carriers were male, while carriers of the ApoE4 allele were 68.1% (*p* = 0.061) male. The vast majority of the sample was of white origin (94.6%), followed by very small minorities of other races, with significant differences observed between the two groups (*p* < 0.0001). When considering the diagnosis, 39.6% of the overall sample was categorized as Cognitively Normal (CN), 44.1% had mild cognitive impairment (MCI) and 16.2% were diagnosed with Dementia. The distribution of diagnoses varied significantly between groups, with ApoE4 carriers showing a higher proportion of dementia cases (27.7% vs. 7.8%) compared to non-carriers (*p* = 0.013). In terms of age, non-carriers were on average 73.02 years old, slightly older than carriers, on average 70.77 years old, (*p* = 0.067). Mean education was approximately 16 years for both groups, with no significant differences (*p* = 0.706). Astrocytic activity evaluated by levels of plasma GFAP was similar between groups, as were whole cerebellar and hippocampal volume values, although the difference in whole cerebellum was borderline statistically significant between ApoE4 carriers and non-carriers (*p* = 0.05) (Table 1).

**Figure 6:**
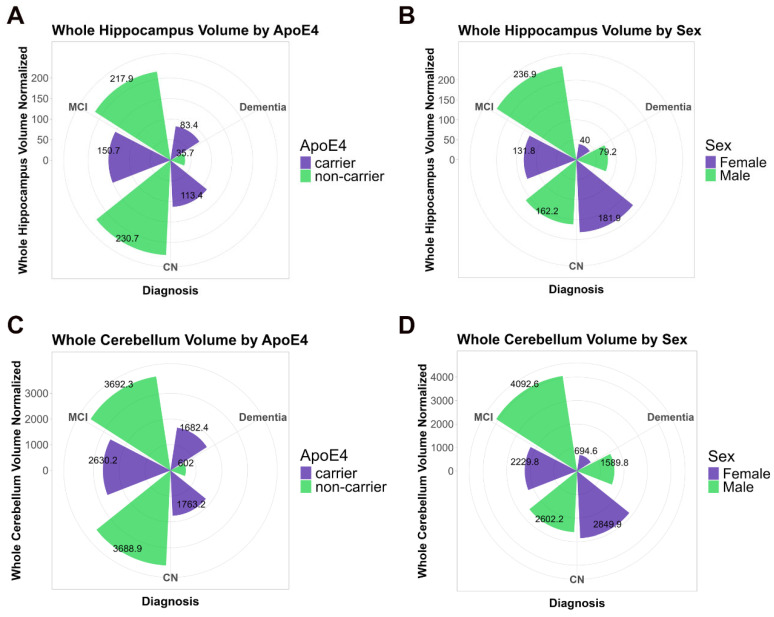
ADNI cohort analysis for Hippocampus and Cerebellum and volume. Circular bar plot shows the total volume for Whole Cerebellum Volume and Total Hippocampal Volume, stratified by ApoE4 status and gender within Diagnostic Categories CN, MCI and AD. (**A**) Whole Cerebellum and (**C**) hippocampus stratified by ApoE4. (**B**) Whole cerebellum and (**D**) hippocampus, stratified by gender.

The total normalized whole cerebellum and hippocampus volumes, stratified by ApoE4 status and sex, respectively, and compared by type of Diagnosis (CN, MCI, and Dementia) are shown in Figure 6A–D to better visualize the variation across diagnostic categories within ApoE4 genotypic groups and between male and female participants.

We conducted multivariate regression analysis with two different models to evaluate the association of GFAP on the whole cerebellum volume and hippocampal volumes, both in the context of ApoE4 status and sex (in Table 2 and Figure 6). In these analyses, intracranial volume was included as a covariate, reflecting its relevance in the examined hippocampal volume models.

**Figure 7:**
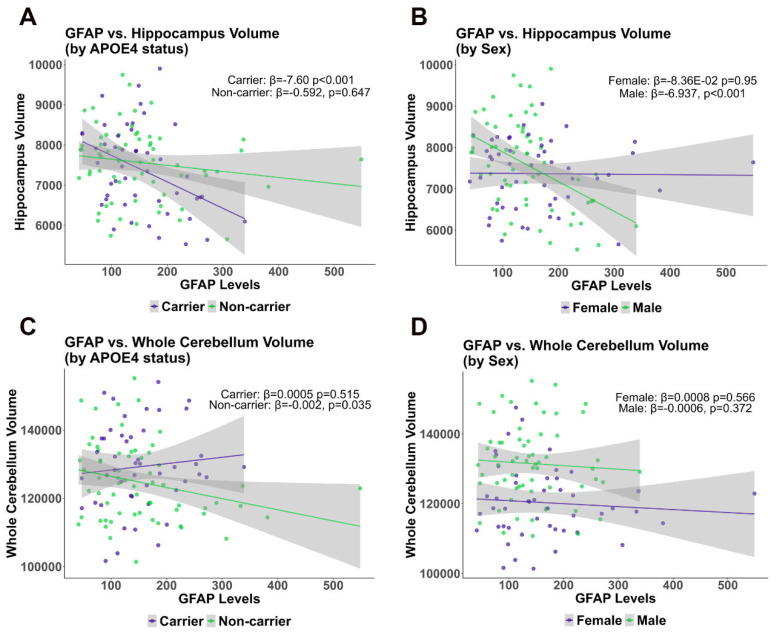
ADNI cohort analysis comparing Cerebellum and Hippocampus volume with GFAP plasma levels. Scatter plot showing the multivariate regression models between GFAP with Whole Cerebellum Volume and Total Hippocampal Volume, stratified by Diagnosis, CN, MCI, and AD in ApoE4 non-carriers and carriers, and males and females, between GFAP and Whole Cerebellum volume. (**A**) Whole Cerebellum and (**C**) hippocampus stratified by ApoE4. (**B**) Whole cerebellum and (**D**) stratified by gender.

According to the adjusted model (Model 2), a significant negative association was observed between GFAP levels and cerebellum volume in ApoE4 non-carriers (β = −0.002046, *p* = 0.04599) but not in ApoE4 carriers (β = 0.0003038, *p* = 0.696). A significant negative association of hippocampal volume with plasma GFAP was found only in ApoE4 carriers (β = −6.504, *p* = 0.00465), while no significant association was observed in non-carriers (β = −0.6497, *p* = 0.61217) (Table 2, Figure 7).

To determine the effect of sex on the association between GFAP levels and volumetric changes, we stratified the analysis by sex. These analyses identified significant negative associations between plasma GFAP and hippocampal volumes for males in the total hippocampus (β = −6.937, *p* = 0.000904) that were ApoE non-carriers, (Model 1 and Model 2), while no significant associations were observed for females, suggesting that in females, plasma GFAP levels failed to predict hippocampal volume (Table 3, Figure 7).

#### 2.1.4. Longitudinal Analysis of Cerebellum and Hippocampus Volume Changes

**Figure 8:**
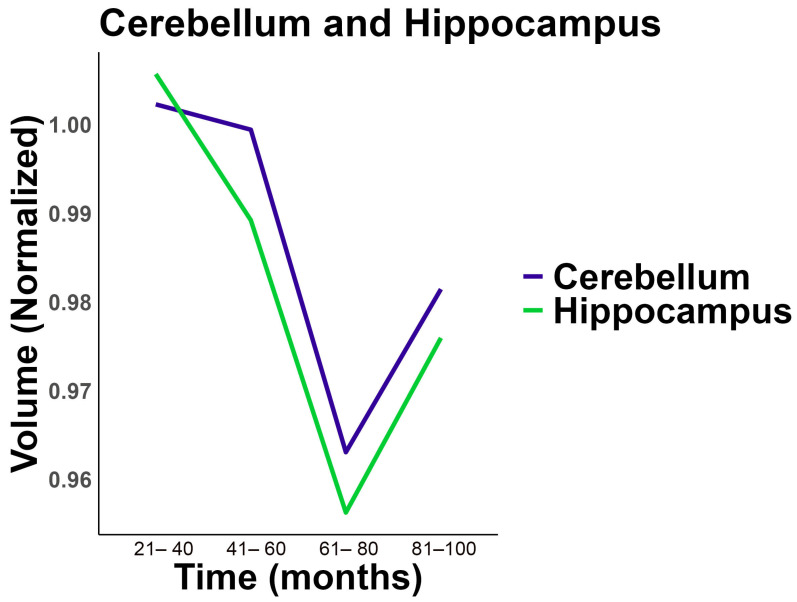
Longitudinal analysis from ADNI cohort comparing cerebellum and hippocampus volume normalized. Distribution of changes in whole cerebellum volume and hippocampus volume over time in longitudinal data from ADNI cerebellum and hippocampal volume.

We conducted longitudinal analysis using linear mixed models to determine whether volume changes were apparent within the same subject over time. We observed a significant decrease in normalized hippocampus and cerebellum volumes across the different time categories (Figure 8). For hippocampus volume, the different times between follow-up visits (month categories), either 41–60 months or 81–100 months, had significantly negative coefficients (*p* < 0.01), suggesting a reduction in volume compared to the reference group (0–20 months). Similarly, for cerebellum volume, the 41–60 months and 81–100 months follow-up categories also showed significantly negative coefficients (*p* < 0.01), indicating a decrease in volume over time (Supplementary Table S1). Likelihood ratio tests confirmed the superiority of the full models over the null models for both volumes, with *p*-values of 0.0015 and 0.0001 for whole hippocampus and cerebellum volumes, respectively, suggesting significant changes over time (Supplementary Table S2).

## 3. Discussion

Most AD-related studies conducted to date have excluded the involvement of the cerebellum in the disease etiology, based on absence of a detectable loss in FDG-PET in AD [39,40]. However, the temporal precision and generalizability of these studies to regional differences during disease progression remained unclear. Consequently, the current study, which detected a delayed change in cerebellum volume and functional changes in an animal model, fills a significant gap in the understanding of AD progression. The cerebellum is often thought to be spared from neurodegenerative processes, but the present findings suggest differently.

Several seminal articles report a role for the cerebellum in the STZ-ICV AD model, wherein it induced deficits including reduced insulin receptor function [41], decreased choline acetyltransferase activity and nerve growth factor levels [42], and increased oxidative stress due to diminished catalase activity [43], collectively indicating a cerebellar involvement in cognitive impairments. However, recent literature lacks temporal and spatial information regarding changes to the cerebellum in this model or in sporadic AD. Here, for the first time, we identified changes in cerebellar volume over time and also considered sex as a biological variable, with an in vivo AD animal model as well as in human cohort studies in ADNI. In addition, we found a temporal association between the hippocampus, which is well documented to be functionally altered in AD and the cerebellum, a structure often underrepresented in experimental investigations in AD. Thus, our study highlights the importance of considering regional vulnerability and temporal dynamics in AD pathophysiology.

Although the cerebellum is one of the last brain regions to show signs of pathology in AD, cerebellar dysfunction has been shown to be associated with cognitive impairment (for a review, see [44]). Besides its central role in motor coordination, the cerebellum is essential for cognition and emotional regulation. However, it is often underrepresented in studies of AD neuropathology as well as learning and behavior. Despite the resistance of the cerebellar tissue to progression of AD pathology, a positive correlation has been reported between the stage of the disease and the cerebellar volume, where smaller cerebellar volume is associated with greater the cognitive decline [45]. Therefore, the reduction in cerebellar volume may be considered a potential indicator of cognitive decline and severity in AD.

**Figure 9:**
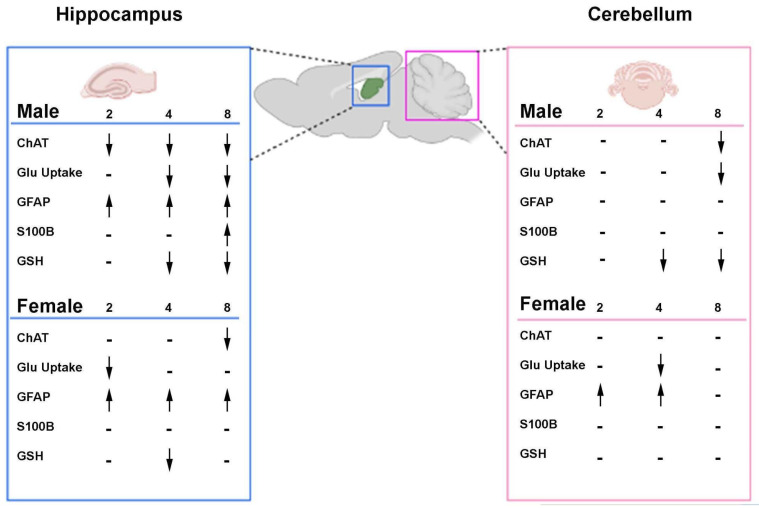
Comparative scheme of parameters evaluated in hippocampus and cerebellum of rats submitted to ICV-STZ infusion. Time is represented in 2, 4, or 8 weeks. Surgery corresponds to STZ or vehicle intracerebroventricular infusion. Original hippocampus study previously published Biasibetti et al. 2017 [32]. Arrows indicate the direction of change over time: ↑ increase, ↓ decrease, and – no significant change, compared to control.

Cerebellar function is dependent on cholinergic signaling (for a review, see [46]). In the STZ-induced AD model, we found choline acetyltransferase (ChAT) levels decreased only in male rats after 8 weeks (Figure 2), corroborating the resistance of the cerebellum to AD pathologies and functional changes as well as complete resistance of females in this model of AD (Figure 9). In vitro, cerebellar granular cells in culture are sensitive to STZ treatment at a concentration sufficient to cause glucose metabolism disturbance [47]. Glucose metabolism, which is compromised in AD and the STZ model, was indirectly measured by glucose uptake in cerebellar slices resulting in a decrease in males only after 8 weeks (Figure 4A). In contrast, females presented an earlier decrease in glucose uptake (4 weeks), which was recovered after 8 weeks (Figure 4B). These results suggest a greater vulnerability of cerebellar cholinergic metabolism in males, which could have implications for sex-specific therapeutic approaches.

In our study, we found a disruption in the main antioxidant defense, GSH, both in male and female STZ-treated animals. However, consistent with other sex dependent effects reported, females presented a delayed effect, and this decline occurred only at 8 weeks (Figure 3A,B). This sexual dimorphism was consistent with our findings in human brain, which revealed that the association of cerebellar volume with plasma GFAP levels was also sex dependent (Figure 6). In the STZ model, GFAP levels increased 2–4 weeks after treatment only in brain tissue from females supporting a differential sex response in astrocyte accumulation. This is temporally concordant with a delay in NOR deficits and the oxidative stress response as well as the resistance to ChAT loss in female cerebellum (Figure 1, Figure 2 and Figure 3). This suggests a role for astrocytes in cerebellum, specifically in female brain and may represent a mechanism of resilience for further studies.

In our previous work, we found sexual dimorphisms in learning and memory were associated with changes in ChAT, GSH, and glucose uptake the hippocampus after STZ-ICV [32]. In our current work, we found sex-dependent differences in the cerebellum after infusion of STZ-ICV in ChAT, GSH, and glucose uptake. Females were resistant to STZ-induced deficits in novel object recognition (Figure 1A) and were resistant to GSH decline (Figure 3B). This greater resistance of female rats to STZ-induced deficits emphasizes the importance of sex-based differences in response to brain injury. In contrast, ChAT levels were unchanged in females, (Figure 2B) and glucose uptake (Figure 4) showed recovery after 8 weeks, again highlighting the sex specific resistance of female cerebellar tissue. These findings emphasize the necessity of incorporating sex as a biological variable in AD research and therapeutic development.

In the STZ model, we found that cerebellar GFAP content increases only in females, suggesting sex specific astrocytic reactivity or protection; however, we did not find S100B alterations in any sex or at any time evaluated (Figure 5). GFAP and S100B represent complementary yet distinct astrocyte markers. GFAP reflects structural activation and reactive astrogliosis, whereas S100B participates in calcium signaling and exerts paracrine effects that can be neurotrophic or neurotoxic depending on its extracellular concentration [48]. Their divergent profiles underscore the complex and context-dependent roles of astrocytes in neurodegeneration. In a diabetes model induced by STZ, the cerebellum of 8 week diabetic mice presented elevated immunostaining for GFAP associated with increased oxidative stress, which was characterized as glial reactivity [49]. In our study, we found resistance to cognitive deficits and cerebellar oxidative stress in females, suggesting that cerebellar GFAP content may be a sign of protection in contrast to reactivity. Notably, in the human context, males exhibited a negative association between hippocampal volume and peripheral GFAP levels, while no such association is observed in females in relation to peripheral GFAP levels (Table 2 and Figure 7). This may be due to the later disease stage in the ADNI data compared to the STZ model. This suggests that GFAP levels may ultimately serve as a sex-dependent biomarker of neurodegeneration in AD.

In the human context, analyses of the ADNI data revealed a negative association between cerebellum volume and peripheral plasma GFAP levels in ApoE4 non-carriers, suggesting a detrimental GFAP response. There was also a negative association between hippocampal volume and plasma GFAP levels; however, only within ApoE4 carriers, suggesting susceptibility to the GFAP response is associated with ApoE4 carriership. Importantly, ApoE is predominantly secreted by astrocytes and has been shown to be involved in neurogenesis [50]. ApoE4 is the major contributor to cholesterol and lipid distribution throughout the brain, suggesting the culpability of astrocyte dysfunction and lipid dyshomeostasis in neurodegeneration. It is important to note that GFAP levels in the plasma may also reflect increased blood–brain barrier permeability, since astrocytes are the primary cells present at the blood–brain barrier unit [51,52]. Although we did not perform GFAP immunostaining in the cerebellum, previous work using the same STZ-ICV model demonstrated increased GFAP expression in the hippocampus through immunohistochemistry [32]. These findings point to astrocytic dysfunction, modulated by APOE genotype, as a central player in regional neurodegeneration.

The present study provides evidence for sexual dimorphism in temporally defined pathophysiology in the cerebellum. Our previous studies have found corresponding results in the hippocampus of the STZ animal model and have been expanded into human studies of hippocampal volume loss in the current report. Here, our observations in the ADNI cohort indicate that the cerebellum is significantly impacted during the preclinical phase of AD (i.e., MCI). Interestingly, males exhibit a larger volume compared to healthy controls (CN), whereas females show a reduction in the cerebellum volume (Figure 6D), in the clinical phase. However, the atrophy rate was higher in AD in both sexes but was associated with APOEe4 status in non-carriers (Table 2). Our longitudinal analysis of hippocampus volume and whole cerebellum volume in the ADNI cohort demonstrate an earlier reduction in hippocampus volume observed between the 21 and 41 month follow-up compared to cerebellum which decreased after the 61–80 month follow-up (Figure 8, Supplementary Tables S1 and S2). This observed biphasic cerebellar volume trajectory may indicate an early phase of reactive changes such as gliosis as compensatory hypertrophy, probably followed by neurodegeneration. Similar phenomena have been described in other models of brain injury and aging [53,54,55], supporting the idea of temporally dynamic glial responses. In addition, previous evidence of network vulnerability of the cerebellum, suggests that AD-related cerebellar atrophy might be secondary to the development of AD pathology in the cerebrum rather than within the cerebellum itself [56]. Because the effect size is only 4%, the clinical significance in relationship to cognition should be further investigated.

The focus on astrocyte biomarkers in AD has been increasing, consistently revealing alterations in AD patients [57]. Previous analyses of the ADNI cohort data showed an independent correlation of plasma GFAP levels with Aβ CSF, but not with tau pathology [58]. Notably, while a robust association between plasma GFAP and Aβ burden appears evident, this association loses its persistence after adjusting for Aβ pathology in relation to tau [16,59,60]. Nonetheless, the production of GFAP by astrocytes and subsequent release or leakage in response to AD pathological events may be displaying sexual dimorphism based on our findings.

Supporting sexual dimorphism, we observed sex-dependent changes in cerebellar volume in our analysis of the ADNI cohort. In our analyses, females showed a visual reduction in cerebellar volume compared to CN, while males had a different pattern, showing an increase in the cerebellar volume at the MCI stage and a decrease in the AD individuals (Figure 6). We know that in humans, AD prevalence is greater in females, but the more severe cases occur in males [61], corroborating our findings.

The final time point in the STZ-ICV-infused model may correspond to a pre-amyloid phase corresponding to MCI in human disease. In the STZ model, alterations in amyloid 42 content and *p*-tau were observed after twenty-four weeks [62], while glucose metabolism alterations and learning and memory deficits occur within two weeks after STZ infusion [32]. The clinical differences observed in the ADNI cohort mirror with the order of the observed deficits in the STZ animal model, highlighting cerebellar involvement, but at a later stage than hippocampal involvement. We have observed that the cerebellum of AD patients is affected by the disease, which may bring into question the suitability of the cerebellum as a reference region for analyzing PET and MRI images, especially at late stages in disease.

Though earlier studies showed cerebellar resistance in AD pathology, the role of the cerebellum in human cognition has been increasingly supported [63]. Functionally, the cerebellum interacts with the limbic system, which is essential for cognition and emotional processing in health and disease [44]. Cerebellar dentate nuclei project to the pedunculopontine nucleus and locus coeruleus, probably modulating acetylcholine and norepinephrine release [64]. Furthermore, developmental studies showed a correlation between a temporally concurrent increase in neuronal number in the cerebrum and cerebellum, supporting a network-specific development of the cerebellum and other brain structures to mediate cognition and movement [65,66,67,68].

One limitation of our study is cross-sectional analysis, because plasma GFAP levels were collected from ADNI participants at a single time point. Emerging longitudinal data as well as emerging plasma biomarkers, including those for neuroinflammation, will ultimately allow a more comprehensive view of the association of GFAP levels with the hippocampus and cerebellum volumetric changes over time.

## 4. Material and Methods

### 4.1. STZ-ICV Animal Model Experiments

#### 4.1.1. Chemicals

Streptozotocin (STZ), 4-(2-hydroxyethyl) piperazine-1-ethane sulfonic acid (HEPES), ophenylenediamine (OPD), o-phthaldialdehyde (OPA), meta-phosphoric acid, sodium nitrate, nitrate reductase, o-phenylenediamine, and monoclonal anti-S100B antibody were purchased from Sigma (St. Louis, MO, USA). Anti-S100 conjugated with peroxidase and anti-GFAP antibodies were from Dako (Santa Clara, CA, USA). Peroxidase secondary antibodies were from Amersham (Stafford, UK). Other reagents were purchased from local commercial suppliers (Sulquímica and Labsul, Porto Alegre, Brazil).

#### 4.1.2. Animals

A total of a hundred and six, three to four-month-old, males and females Wistar rats weighing 300–400 g were obtained from our breeding colony (at the Department of Biochemistry, Federal University of Rio Grande do Sul, Porto Alegre, Brazil). The animals were maintained under controlled light and environmental conditions 12 h light/dark cycle at a temperature of 24 ± 2 °C, 50–60% relative humidity, and were housed in groups of four in standard cages with ad libitum access to drinking water and standard food pellets. All animal procedures were performed according to guidelines of the National Institutes of Health Guide for the Care and Use of Laboratory Animals, and all protocols were approved by the Federal University of Rio Grande do Sul Animal Care and Use Committee (process number 30626). Rats were divided into 2 groups: sham and STZ.

#### 4.1.3. Surgical Procedure

STZ was ICV-infused, based on previous studies [32,69,70]. Briefly, on the day of the surgery, animals were anesthetized with ketamine/xylazine (75 and 10 mg/kg, respectively, i.p.) and placed in a stereotaxic apparatus. A midline sagittal incision was made in the scalp. Burr holes were drilled in the skull on both sides over the lateral ventricles. The lateral ventricles were accessed using the following coordinates: 0.9 mm posterior to bregma; 1.5 mm lateral to sagittal suture; 3.6 mm beneath the brain’s surface. Rats received a single bilateral infusion of 5 μL STZ (3 mg/kg) or Sham group with vehicle (Hank’s balanced salt solution—HBSS—containing in mM 137 NaCl; 0.63 Na_2_HPO_4_; 4.17 NaHCO_3_; 5.36 KCl; 0.44 KH_2_PO_4_; 1.26 CaCl_2_; 0.41 MgSO_4_; 0.49 MgCl_2_ and 10 glucose, in pH 7.4) using a Hamilton syringe. After the surgical procedure, rats were placed on a heating pad to maintain body temperature at 37.5 ± 0.5 °C and kept there until recovery from anesthesia. The animals were submitted to behavioral tasks and biochemical analysis at each time point 2, 4, and 8 weeks after STZ injection. Animals were euthanized, and brains collected 12 h after the final test session to reduce the influence of acute behavioral stress on molecular outcomes.

#### 4.1.4. The Novel Object Recognition Test (NOR)

The NOR test is performed during the rat’s active cycle (dark phase) and consists of three phases: habituation, sample, and test performed in an open-field apparatus (50 cm sides). For habituation, animals are placed in the lateral area of the apparatus and allowed to explore the open-field arena freely, in the absence of objects, for 10 min. It is ensured that the rats actively explore the arena to better focus on objects in the next step. Twenty-four hours after the habituation phase, the animals undergo the sample phase: the rat is returned to the apparatus that contains two identical and previously unknown sample objects (A + A). After 24, following the sample phase, the rats return to the apparatus to test long-term (LTM) memory, respectively. During the sample phase, it is crucial to ensure that the animal has no preference for one of the objects, making a symmetric exploration of both. In the test session, the rat is returned to the open-field arena containing two objects; one object is identical to the sample session (familiar object), and the other is novel and previously unknown (A + C). Usually, our chosen recognition index is calculated as follows: time exploring the novel object/time exploring both objects preference index percentage (Index%). Exploration is defined as sniffing or touching the object with the nose and/or forepaws. The apparatus and the objects are thoroughly cleaned with 70% ethanol between trials to ensure the absence of olfactory cues [32,71,72,73]. It is important not to leave any residue of alcohol on objects. All the steps for each rat are recorded and the footage is further analyzed by at least two “blind” observers.

#### 4.1.5. ELISA for S100B and GFAP

Cerebellum S100B content was measured by ELISA [74]. Briefly, 50 μL of the sample plus 50 μL of Tris buffer were incubated for 2 h on a microtiter plate previously coated with monoclonal anti-S100B (SH-B1). Polyclonal anti-S100B was incubated for 30 min, and then peroxidase-conjugated anti-rabbit antibody was added for a further 30 min. A colorimetric reaction with o-phenylenediamine was measured at 492 nm. The standard S100B curve ranged from 0.020 to 10 ng/mL. The ELISA for Cerebellum GFAP [75] was carried out by coating the microtiter plate with 100 μL samples containing 30 μg of protein for 24 h at 4 °C. Incubation with a rabbit polyclonal anti-GFAP for 2 h was followed by incubation with a secondary antibody conjugated with peroxidase for 1 h, at room temperature; the standard GFAP curve ranged from 0.1 to 10 ng/mL.

#### 4.1.6. Quantification of Glutathione

The glutathione content was determined according to [76]. The cerebellar slices were homogenized in a phosphate-KCl buffer (20–140 mM, pH 7.4) containing 5 mM EDTA, and the proteins were precipitated with the addition of 1.7% meta-phosphoric acid. The supernatant was collected and o-phthalialdehyde (1 mg/mL in methanol) was added at room temperature for 15 min. Fluorescence was measured using the excitation and emission lengths of 350 and 420 nm, respectively. A calibration curve was made with a standard glutathione solution (0–500 μM).

#### 4.1.7. Glucose Uptake

Glucose uptake was performed as previously described [77,78], with some modifications. The 300 µm cerebellar slices were transferred to a 24-well plate and incubated for 15 min at 35 °C in Hank’s balanced salt solution (HBSS). The assay started with adding 0.1 μCi/well D-[2,3-^3^H] deoxy-glucose for 30 min. The incubation was stopped by removing the medium and rinsing the slices twice with ice-cold HBSS. The slices were then lysed in a 0.5 M NaOH solution. Glucose uptake was calculated by subtracting the nonspecific uptake obtained using the glucose transporter inhibitor, cytochalasin B (25 μM), from the total uptake. Radioactivity was measured using a scintillation counter. Results are expressed as nmol/mg protein/min.

#### 4.1.8. Western Blot Analysis for ChAT

Proteins in the samples were homogenized in sample buffer (62.5 mM Tris–HCl, pH 6.8, 10% (*v*/*v*) glycerol, 2% (*w*/*v*) SDS, 5% (*w*/*v*) β-mercaptoethanol, and 0.002% bromphenol blue) and separated by SDS-PAGE on 12% (*w*/*v*) acrylamide gel before electro transferring onto nitrocellulose membranes. The membranes were blocked with 2% chicken egg in tris-buffered saline with Tween 20 (TTBS) (20 mmol/L Tris–HCl, pH 7.5, 137 mmol/L NaCl, 0.05% (*v*/*v*) Tween 20) and then incubated overnight (4 °C). Subsequently, the membranes were incubated overnight with the appropriate primary antibodies: ChAT (1:5000) EMD Millipore (Burlington, MA, USA), e β-actin (1:2000) Sigma Aldrich (St. Louis, MO, USA), in TTBS and 1% bovine standard albumin; BSA. Next, the membranes were incubated for 1 h at room temperature with a secondary antibody, horseradish peroxidase (HRP)-conjugated anti-mouse IgG (1:10,000, Dako, Santa Clara, CA, USA). Actin (Millipore; Darmstadt, Germany) was used as a loading control. Chemiluminescent bands were detected using Imagequant LAS4000 1.2 GE Healthcare (Chicago, IL, USA), and densitometric analyses were performed using Image-J Java 8 software. Results are expressed as percentages of the control.

#### 4.1.9. Protein Determination

Protein content was measured by Lowry’s method with some modifications, using bovine serum albumin as the standard [79].

### 4.2. Statistical Analysis

For the animal study, all results were presented as mean ± standard error for each time point, assuming *p* < 0.05. The data were analyzed by Student’s *t*-test, and for the OR test, data were statistically evaluated by One sample *t*-test difference of 50%. For STZ gender-related differences over time, we performed two-way ANOVA followed by Sidak’s test. All analyses were performed using GraphPad Prism software version 8 (La Jolla, CA, USA).

#### 4.2.1. Alzheimer’s Disease Neuroimaging Initiative (ADNI)

##### Florbetapir PET Amyloid Imaging

Since the advent of long-lived Florbetapir imaging-labeled positron emitting agents for amyloid imaging, it has become possible to assess brain amyloid accumulation in three ADNI cohorts: older individuals without cognitive impairment at baseline and those with mild cognitive impairment or Alzheimer’s disease. Amyloid PET scans with mean standard uptake ratios (SUVR) equal to or greater than 1.17 have already been performed and described above. Each ADNI participant was given an intravenous bolus injection of AV-45 containing 370 MBq (10 mCi ±10%). After an interval of about 50 min for drug action, a continuous 20 min PET brain imaging session was performed, segmented into four 5 min intervals, to collect dynamic amyloid data (The Alzheimer’s Disease Neuroimaging Initiative positron emission tomography core).

##### MRI—FreeSurfer Methods

The FreeSurfer Image Analysis Suite is used for cortical reconstruction and volumetric segmentation (http://surfer.nmr.mgh.harvard.edu/), accessed on 19 July 2024. Processing includes motion correction and data averaging, as per Reuter et al., especially when multiple T1-weighted images are available [80]. It also encompasses the removal of non-brain tissue through hybrid procedures, as described by Segonne et al. 2004, in addition to automatic Talairach transformation, and segmentation of subcortical white matter and deep gray matter volumetric structures [81]. The latter include the hippocampus, amygdala, caudate, putamen, and ventricles, as detailed by Fischl et al. in several works between 2002 and 2004 [82].

The process also includes intensity normalization [83], delimitation between gray and white matter, automatic topological correction, and surface deformation following intensity gradients. The goal is to optimally position the boundaries between fabrics, as evidenced by Dale and collaborators between 1993 and 1999 [84].

##### Statistical Analysis

Data were analyzed with the following statistical methods and absolute values, percentages, means, and standard deviations are reported. The chi-square test was used for categorical variables, while the *t*-test was used for continuous variables, assuming a normal distribution as confirmed by the Shapiro–Wilk test. We constructed multivariate linear models to determine whether GFAP and other variables are associated with hippocampus and cerebellum volume outcomes. This information was illustrated in detail using tables and scatter plots. A linear mixed model was applied to assess the longitudinal change in normalized hippocampus and cerebellum volumes across different time categories. The normalized volumes were obtained by dividing the original volume by the mean volume of the respective region (cerebellum or hippocampus). The variable Time Category was used as a fixed effect, categorizing participants into time ranges (0–20, 41–60, 61–80, 81–100 months), and a random effect for the participant identifier was included to account for individual variability. To minimize bias, clinically relevant variables were considered when adjusting the models. The significance level was set at 5%. As an analysis platform, R software version 4.2.2 was used as well as the ggplot2 package for graphical representation.

## 5. Conclusions

Our results emphasize the importance of temporally coordinated sex-specific and regional changes in AD, which depend on ApoE status. We found that plasma GFAP levels are negatively associated with both cerebellar and hippocampus volume during AD progression. This can be due to functional networks as well as astrocyte infiltration, which may be independent of and possibly preceded, primary pathology. We found ordinal concordance between the ADNI studies and the animal model, which both showed earlier hippocampal deficits compared to delayed cerebellar functional and structural deficits. In conclusion, we have identified an association between plasma GFAP and both cerebellum and hippocampus volumes in the human brain and a concordant temporally dependent involvement of the cerebellum both in human brain and in the STZ sAD-type model. These differences were dependent on sex as well as sensitive to ApoE genotype, emphasizing the importance of these sub-group analyses in future studies of the potential culpability of regional astrocyte function in AD physiopathology and disease progression.

## Figures and Tables

**Table 1:** Demographics information of sample from ADNI study with GFAP information.

	Overall	ApoE4 Non-Carrier	ApoE4 Carrier	*p*-Value *
N	111	64	47	
	N (%)	N (%)	N (%)	
Sex = Male	63 (56.8)	31 (48.4)	32 (68.1)	0.061
Race				<0.0001
Non-whites	6 (5.4)	5 (7.9)	1 (2.1)	
Whites	105 (94.6)	59 (92.1)	46 (97.9)	
	Mean (SD)	Mean (SD)	Mean (SD)	
Diagnosis				0.013
CN	44 (39.6)	30 (46.9)	14 (29.8)	
MCI	49 (44.1)	29 (45.3)	20 (42.6)	
Dementia	18 (16.2)	5 (7.8)	13 (27.7)	
Age	72.07 (6.36)	73.02 (5.94)	70.77 (6.75)	0.067
Education (years)	16.31 (2.73)	16.39 (2.55)	16.19 (2.98)	0.706
GFAP	152.15 (81.26)	151.82 (91.74)	152.62 (65.30)	0.959
Whole Cerebellum (SUVR)	126,657.09 (12,057.85)	124,737.36 (11,382.75)	129,271.19 (12,574.43)	0.05
Hippocampus volume (mm^3^)	7494.86 (984.53)	7568.33 (903.69)	7394.83 (1087.06)	0.361

* *p*-value was used to compare the differences between ApoE 4 non-carriers and carriers, employing a *t* test for continuous variables and a chi square test for categorical variables.

**Table 2:** Association between GFAP and brain areas, stratified by status of ApoE4.

		ApoE4 Non-Carrier		ApoE4 Carrier	
Predictor	Outcome	β	*p*-Value	β	*p*-Value
		Model 1			
GFAP	Whole Cerebellum Vol. SUVR	−0.002124	0.0354	0.0005056	0.515
	Total Hippocampus vol (mm^3^)	−0.5919	0.64735	−7.598	0.00097
		Model 2			
GFAP	Whole Cerebellum Vol. SUVR	−0.002046	0.04599	0.0003038	0.696
	Total Hippocampus vol (mm^3^)	−0.6497	0.61217	−6.504	0.00465

Association analysis using Multivariate Regression Models. M1: Model crude; M2: Model adjusted by Diagnosis. In the Hippocampus analysis intracranial volume was included in all models as a covariate.

**Table 3:** Association between GFAP and brain areas, stratified by Sex.

		Male		Female	
Predictor	Outcome	β	*p*-Value	β	*p*-Value
		Model 1			
GFAP	Whole Cerebellum Vol. SUVR	−0.0006723	0.3718	−0.0008029	0.566
	Total Hippocampus vol (mm^3^)	−6.937	0.000904	−8.36 × 10^−2^	0.949968
		Model 2			
GFAP	Whole Cerebellum Vol. SUVR	−0.0006808	0.3701	−0.000828	0.563
	Total Hippocampus vol (mm^3^)	−6.721	0.000942	0.06878	0.958013

Association analysis using Multivariate Regression Models. M1: Model adjusted by ApoE4 status; M2: Model adjusted by ApoE4 status and Diagnosis. In the Hippocampus analysis, intracranial volume in all models was included as a covariate.

## Data Availability

Data used in preparation of this article was obtained from the Alzheimer’s Disease Neuroimaging Initiative (ADNI) database (https://adni.loni.usc.edu). As such, the investigators within the ADNI contributed to the design and implementation of ADNI and/or provided data but did not participate in analysis or writing of this report. A complete listing of ADNI investigators can be found at: http://adni.loni.usc.edu/wp-content/uploads/how_to_apply/ADNI_Acknowledgement_List.pdf. Accessed on 19 July 2024.

## Supplementary Materials

The following supporting information can be downloaded at: https://www.mdpi.com/article/10.3390/ijms26104810/s1.
