# Supplementary material for: Sex-Associated Cerebellar and Hippocampal Volume Reduction in Alzheimer’s Disease: Insights from the Clinical ADNI Cohort and STZ Animal Model
Source: Int J Mol Sci. 2025 May 17;26(10):4810. doi: 10.3390/ijms26104810 (PMC12112559; doi:10.3390/ijms26104810)
Supplement: Supplementary file 1 [file ijms-26-04810-s001.zip › ijms-3598726-supplementary.pdf]

Supplementary Table S1. Longitudinal analysis of normalized hippocampus and cerebellum volumes across time categories using linear mixed-effects models.

Region	Time Category	Beta Coefficient	Std. Error	t-value	Significance
Hippocampus	41–60 months	-0.02197	0.005632	-3.901	p < 0.001
Hippocampus	61–80 months	-0.053999	0.021455	-2.517	p < 0.05
Hippocampus	81–100 months	-0.063429	0.016527	-3.838	p < 0.001
Cerebellum	41–60 months	-0.00765	0.002464	-3.105	p < 0.01
Cerebellum	61–80 months	-0.012214	0.009556	-1.278	n.s.
Cerebellum	81–100 months	-0.024273	0.007365	-3.296	p < 0.01

Beta coefficients, standard errors, and t-values are presented for each time category compared to the reference group (0–20 months). Models were adjusted for intra-subject variability (random intercept for subject ID). Intracranial volume was included as a covariate in hippocampus models. p-values are reported.

Supplementary Table S2. Likelihood ratio tests were used to compare full linear mixed models (including time category) with null models (random intercept only) for each brain region. The hippocampus model included intracranial volume as a covariate.

Region	Model Compared	Chi-square	Degrees of Freedom	p-value
Hippocampus	Full vs Null	20.377	3	0.00015
Cerebellum	Full vs Null	15.427	3	0.001

Chi-square statistics, degrees of freedom, and p-values from likelihood ratio tests comparing full models (with time category) to null models (random intercept only) for each brain region. The hippocampus model was adjusted for intracranial volume.
